# Supplementary material for: Mesoscale phase separation of chromatin in the nucleus
Source: eLife. 2021 May 4;10:e63976. doi: 10.7554/eLife.63976 (PMC8139833; doi:10.7554/eLife.63976)
Supplement: Transparent reporting form [file elife-63976-transrepform.pdf]

eLIFE
1st Floor
24 Hills Road
Cambridge CB2 1JP, UK
P 01223 855340
W elifesciences.org
T @elife

eLife's transparent reporting form

We encourage authors to provide detailed information *within their submission* to facilitate the interpretation and replication of experiments. Authors can upload supporting documentation to indicate the use of appropriate reporting guidelines for health-related research (see EQUATOR Network), life science research (see the BioSharing Information Resource), or the ARRIVE guidelines for reporting work involving animal research. Where applicable, authors should refer to any relevant reporting standards documents in this form.

If you have any questions, please consult our Journal Policies and/or contact us: editorial@elifesciences.org.

Sample-size estimation

- You should state whether an appropriate sample size was computed when the study was being designed
- You should state the statistical method of sample size computation and any required assumptions
- If no explicit power analysis was used, you should describe how you decided what sample (replicate) size (number) to use

Please outline where this information can be found within the submission (e.g., sections or figure legends), or explain why this information doesn't apply to your submission:

This paper is a coarse grained simulation study of peripheral, central, and conventional organization of chromatin in the nucleus. We provide a list of model parameters and their values in Table 1 of supplementary information (SI). We compared our simulation results with experiments on intact-organism imaging of Drosophila larvae. Experimental documentation techniques, sample sizes etc. are described in our experimental paper titled "Live imaging of chromatin distribution in muscle nuclei reveals novel principles of nuclear architecture and chromatin compartmentalization" whose preprint is published on the server https://doi.org/10.1101/2020.06.21.163360.

Replicates

- You should report how often each experiment was performed
- You should include a definition of biological versus technical replication
- The data obtained should be provided and sufficient information should be provided to indicate the number of independent biological and/or technical replicates
- If you encountered any outliers, you should describe how these were handled
- Criteria for exclusion/inclusion of data should be clearly stated
- High-throughput sequence data should be uploaded before submission, with a private link for reviewers provided (these are available from both GEO and ArrayExpress)

Please outline where this information can be found within the submission (e.g., sections or figure legends), or explain why this information doesn't apply to your submission:

eLife Sciences Publications, Ltd is a limited liability non-profit non-stock corporation incorporated in the State of Delaware, USA, with company number 5030732, and is registered in the UK with company number FC030576 and branch number BR015634 at the address 1st Floor, 24 Hills Road, Cambridge CB2 1JP | August 2014

The main focus of this paper is the conceptual understanding of the experiments (shown in the SI of our manuscript) whose details are given in a separate paper with a preprint published on: https://doi.org/10.1101/2020.06.21.163360.
Simulation part: To show the transition from peripheral to conventional to central organizations of chromatin, we performed multiple independent Brownian Dynamics simulations changing the parameters of hydration, chromatin-lamina interactions, and intra-chromatin interactions.
Experiment part: Experiment's details are described in our experimental paper titled "Live imaging of chromatin distribution in muscle nuclei reveals novel principles of nuclear architecture and chromatin compartmentalization" whose
preprint is published on the server doi: https://doi.org/10.1101/2020.06.21.163360.

Statistical reporting

- Statistical analysis methods should be described and justified
- Raw data should be presented in figures whenever informative to do so (typically when N per group is less than 10)
- For each experiment, you should identify the statistical tests used, exact values of N, definitions of center, methods of multiple test correction, and dispersion and precision measures (e.g., mean, median, SD, SEM, confidence intervals; and, for the major substantive results, a measure of effect size (e.g., Pearson's r, Cohen's d)
- Report exact p-values wherever possible alongside the summary statistics and 95% confidence intervals. These should be reported for all key questions and not only when the p-value is less than 0.05.

Please outline where this information can be found within the submission (e.g., sections or figure legends), or explain why this information doesn't apply to your submission:

Fig.2, Fig.3, Fig.4 – Chromatin concentrations and volume fractions profile are calculated by volmap and rdf function of VMD software. The quantities were computed for 25 different configurations (from the last 5 millisecond of a 120 millisecond simulation run).
Fig.1 and Fig.6 (a) (b) – 3D configurations of chromatin are calculated by VMD software visualizing last trajectory of simulation.
Fig.5 – State diagrams: From the plots of local volume fraction, we classified the chromatin organization as peripheral, central or conventional.
Fig.6 (c) – Experimental figure details are described in our experimental paper titled "Live imaging of chromatin distribution in muscle nuclei reveals novel principles of nuclear architecture and chromatin compartmentalization" whose
preprint is published on the server doi: https://doi.org/10.1101/2020.06.21.163360.

eLife Sciences Publications, Ltd is a limited liability non-profit non-stock corporation incorporated in the State of Delaware, USA, with company number 5030732, and is registered in the UK with company number FC030576 and branch number BR015634 at the address 1st Floor, 24 Hills Road, Cambridge CB2 1JP | August 2014

(For large datasets, or papers with a very large number of statistical tests, you may upload a single table file with tests, Ns, etc., with reference to sections in the manuscript.)

Group allocation

- Indicate how samples were allocated into experimental groups (in the case of clinical studies, please specify allocation to treatment method); if randomization was used, please also state if restricted randomization was applied
- Indicate if masking was used during group allocation, data collection and/or data analysis

Please outline where this information can be found within the submission (e.g., sections or figure legends), or explain why this information doesn't apply to your submission:

This study employs coarse-grained simulations and hence there is no group allocation of samples. We compared our simulation results with experimental results. Experimental details are described in our experimental paper titled "Live imaging of chromatin distribution in muscle nuclei reveals novel principles of nuclear architecture and chromatin compartmentalization" whose preprint is published on the server doi: https://doi.org/10.1101/2020.06.21.163360.

Additional data files ("source data")

- We encourage you to upload relevant additional data files, such as numerical data that are represented as a graph in a figure, or as a summary table
- Where provided, these should be in the most useful format, and they can be uploaded as "Source data" files linked to a main figure or table
- Include model definition files including the full list of parameters used
- Include code used for data analysis (e.g., R, MatLab)
- Avoid stating that data files are "available upon request"

Please indicate the figures or tables for which source data files have been provided:

We modeled the 22.4 Mbp regions of chromosome X (ChrX) of Drosophila where the distribution of the LAD regions is available online server http://compbio.med.harvard.edu/modencode/webpage/lad/fly.kc.DamID.dm3.bed.
We also used a Monte-Carlo method to randomly distribute the LAD regions along the chromatin chain. We used LAMMPS software to run our simulation and generate raw data of 3D configurations of chromatin. From the raw data of 3D configuration, we used VMD and MATLAB software to calculated statistical quantities such as chromatin concentrations and local volume fraction profile. In Table 1 of SI, we outline the values of physical parameters used in our simulations.
